# Supplementary figures and images for: Genome-wide analysis of PRR gene family uncovers their roles in circadian rhythmic changes and response to drought stress in Gossypium hirsutum L
Source: PeerJ. 2020 Sep 25;8:e9936. doi: 10.7717/peerj.9936 (PMC7521341; doi:10.7717/peerj.9936)

A

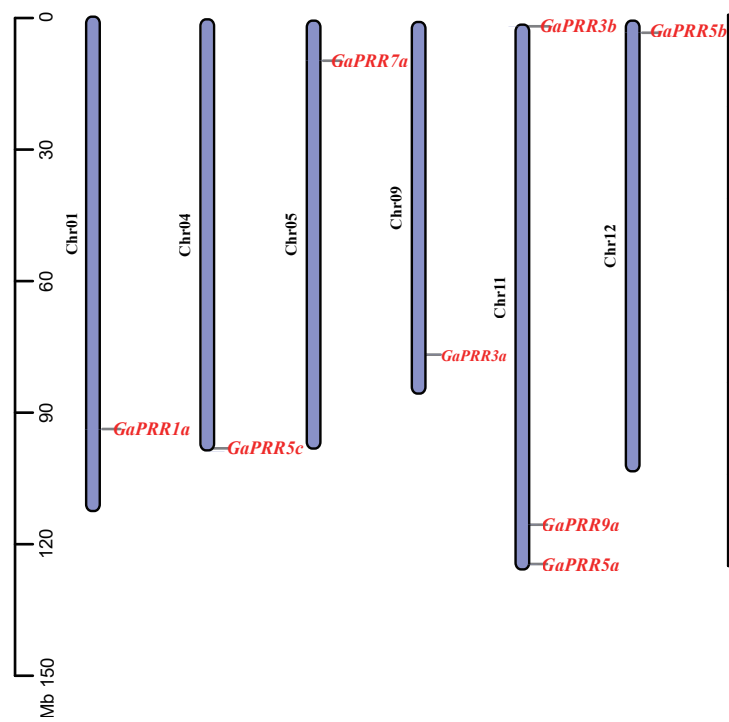

B

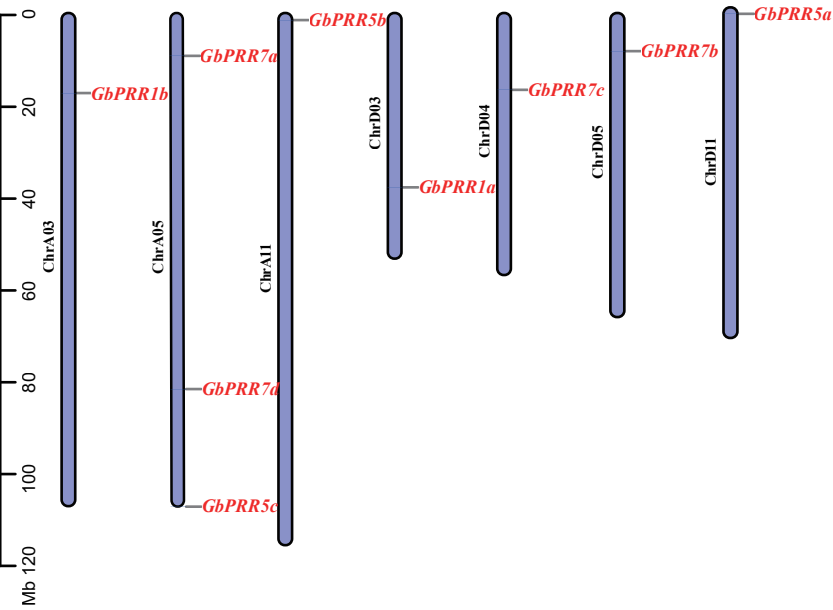

C

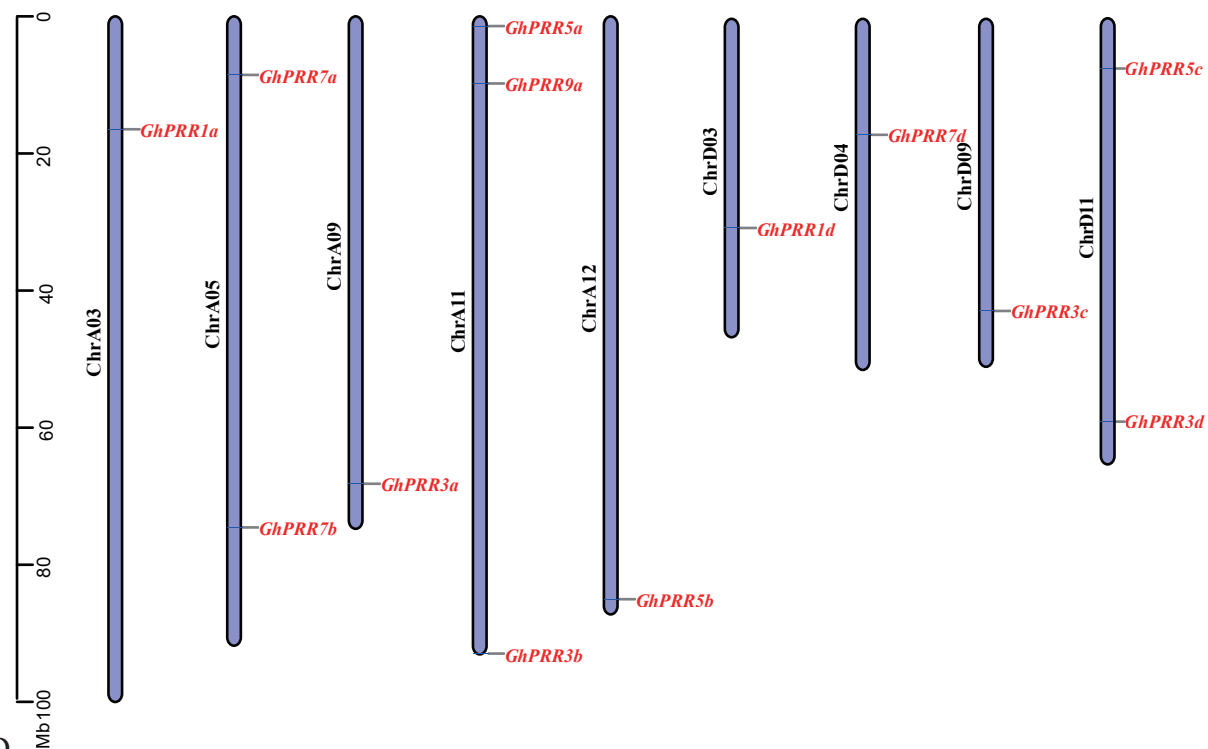

D

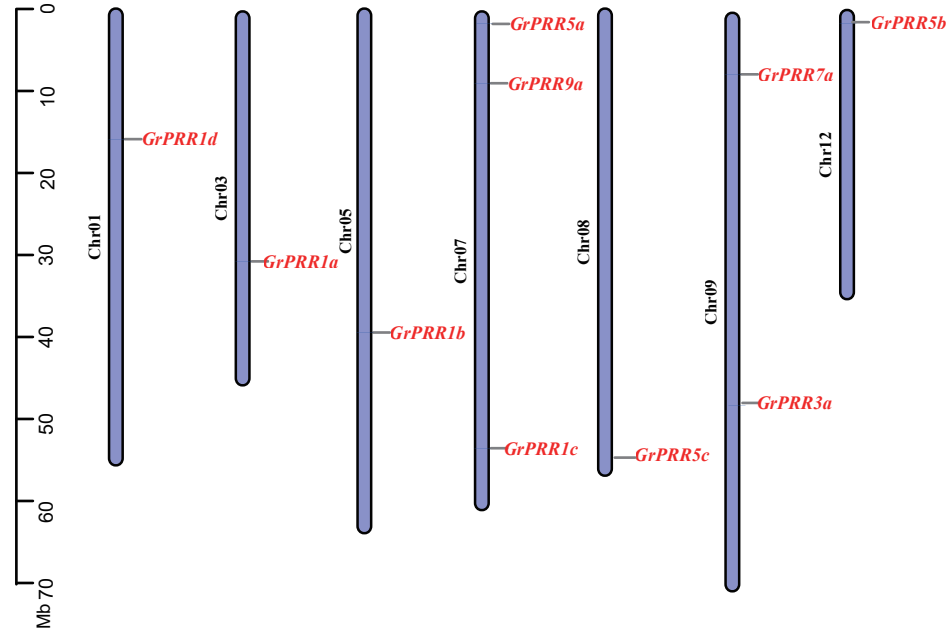

Supplement: Supplemental Information 1 — (A) Distributions of GaPRR genes on chromosomes in G. arboretum; (B) Distributions of GbPRR genes on chromosomes in G. barbadense; (C) Distributions of GhPRR genes on chromosomes in G. hirsutum; (D) Distributions of GrPRR genes on chromosomes in G. raimondii. The chromosome number is shown above each chromosome. The scale bar beside the chromosome indicates the length in mega-bases (Mb). [file peerj-08-9936-s001.pdf]

**Cluster 1**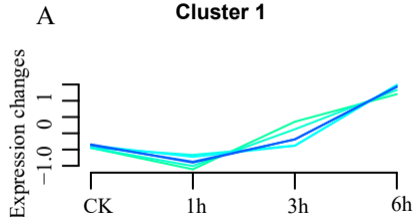**Cluster 2**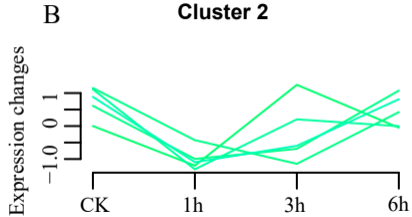**Cluster 3**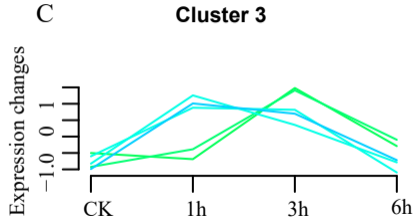

Supplement: Supplemental Information 2 [file peerj-08-9936-s002.pdf]

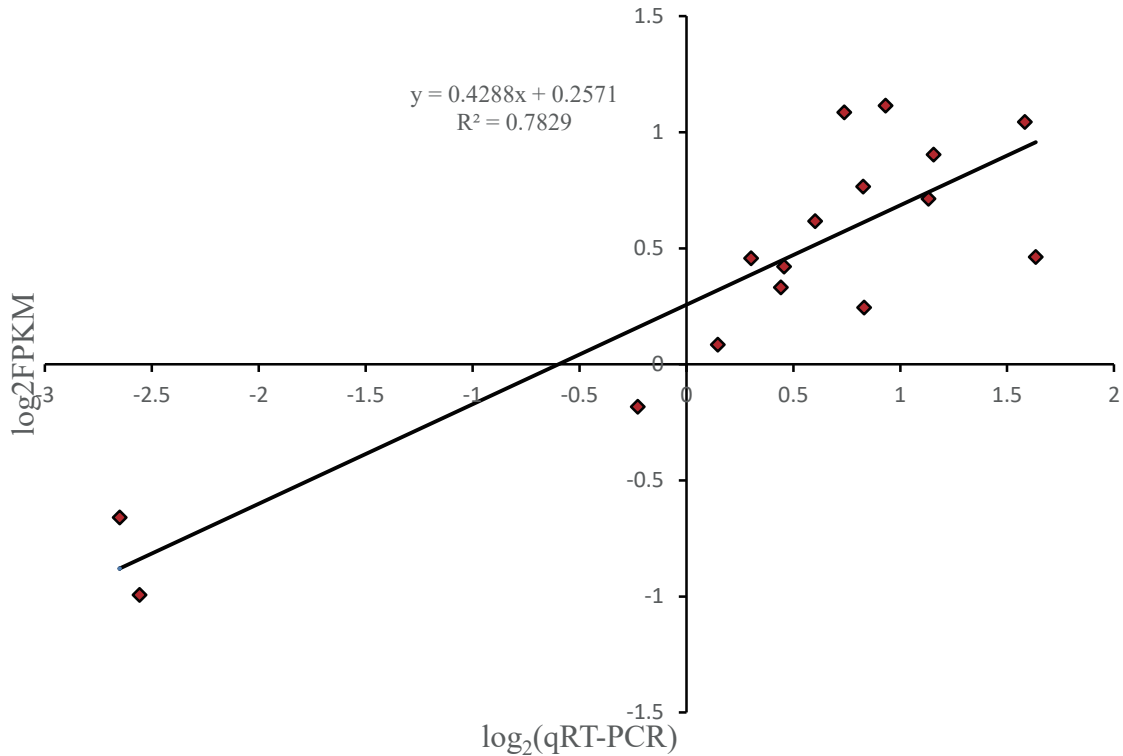

Supplement: Supplemental Information 3 — Scatter plots represent log2 expression ratios calculated from qRT-PCR and RNA-seq of GhPRR genes. The relative expression value from qRT-PCR; X-axis: the FPKM value from transcriptomic data sets. [file peerj-08-9936-s003.pdf]
